# A Protein-Conjugate Approach to Develop a Monoclonal Antibody-Based Antigen Detection Test for the Diagnosis of Human Brucellosis

**DOI:** 10.1371/journal.pntd.0002926

**Published:** 2014-06-05

**Authors:** Kailash P. Patra, Mayuko Saito, Vidya L. Atluri, Hortensia G. Rolán, Briana Young, Tobias Kerrinnes, Henk Smits, Jessica N. Ricaldi, Eduardo Gotuzzo, Robert H. Gilman, Renee M. Tsolis, Joseph M. Vinetz

**Affiliations:** 1 Division of Infectious Diseases, Department of Medicine, University of California San Diego, La Jolla, California, United States of America; 2 Department of Medical Microbiology, University of California Davis, Davis, California, United States of America; 3 Department of Biomedical Research, Royal Tropical Institute, Amsterdam, the Netherlands; 4 Institute of Tropical Medicine Alexander von Humboldt, Universidad Peruana Cayetano Heredia, Lima, Peru; 5 Department of International Health, Johns Hopkins Bloomberg School of Public Health, Baltimore, Maryland, United States of America; 6 Laboratory of Research and Development, and Department of Cellular and Molecular Sciences, Faculty of Sciences, Universidad Peruana Cayetano Heredia, Lima, Peru; University of Tennessee, United States of America

## Abstract

Human brucellosis is most commonly diagnosed by serology based on agglutination of fixed *Brucella abortus* as antigen. Nucleic acid amplification techniques have not proven capable of reproducibly and sensitively demonstrating the presence of *Brucella* DNA in clinical specimens. We sought to optimize a monoclonal antibody-based assay to detect *Brucella melitensis* lipopolysaccharide in blood by conjugating *B. melitensis* LPS to keyhole limpet hemocyanin, an immunogenic protein carrier to maximize IgG affinity of monoclonal antibodies. A panel of specific of monoclonal antibodies was obtained that recognized both *B. melitensis* and *B. abortus* lipopolysaccharide epitopes. An antigen capture assay was developed that detected *B. melitensis* in the blood of experimentally infected mice and, in a pilot study, in naturally infected Peruvian subjects. As a proof of principle, a majority (7/10) of the patients with positive blood cultures had *B. melitensis* lipopolysaccharide detected in the initial blood specimen obtained. One of 10 patients with relapsed brucellosis and negative blood culture had a positive serum antigen test. No seronegative/blood culture negative patients had a positive serum antigen test. Analysis of the pair of monoclonal antibodies (2D1, 2E8) used in the capture ELISA for potential cross-reactivity in the detection of lipopolysaccharides of *E. coli* O157:H7 and *Yersinia enterocolitica* O9 showed specificity for *Brucella* lipopolysaccharide. This new approach to develop antigen-detection monoclonal antibodies against a T cell-independent polysaccharide antigen based on immunogenic protein conjugation may lead to the production of improved rapid point-of-care-deployable assays for the diagnosis of brucellosis and other infectious diseases.

## Author Summary

Brucellosis is a OneHealth disease reflecting the risk for human infection by interaction with and relation to affected animal populations. The disease is often difficult to diagnose because of lack of precise or accessible diagnostic reagents, and because culture is complex, hazardous and relatively insensitive. Brucellosis disproportionately affects the poor and dispossessed with human and animal burdens of disease in the Middle East, North Africa, Mongolia and other regions that are simply unknown. The diagnosis of brucellosis most often rests on serological tests—antibody detection—based on agglutination of fixed *Brucella abortus*. We have developed the basis for developing a new test based on the detection of the *B. melitensis* lipopolysaccharide, which provides rapid and definitive identification of the presence of the organism in clinically obtainable body fluids. A new approach—protein conjugation to the lipopolysaccharide antigen—was taken to enhance the affinity of the monoclonal antibodies that were generated for the test. These reagents were tested in a mouse model of *B. melitensis* and in humans from the brucellosis-endemic region of Peru, and provided the data for the basis of further clinical development and clinical trials for the rapid, point-of-care diagnosis of brucellosis that will also provide new tools for assessing the global burden of disease.

## Introduction

Human brucellosis is most commonly caused by two species of the genus *Brucella*, typically *B. abortus* from cattle and *B. melitensis* from goats and sheep. The definitive diagnosis of brucellosis rests upon demonstration of the causative bacterium in a suspected patient's body fluid, typically by culture isolation [1], [2]. While detection of *Brucella* nucleic acids [3]–[13] or antigens [14] would be expected to be diagnostic for new cases of brucellosis, DNA has been reported to persist in blood after successful treatment of solidly diagnosed cases [15], [16]. Therefore PCR amplification-based tests are not useful to confirm brucellosis relapse [15], [16]. Because culture is technically challenging and hazardous in many clinical laboratories, brucellosis is most commonly diagnosed using serological methods that use fixed, whole *Brucella abortus* as antigen [17]–[21]. Such methods include the Rose Bengal, slide agglutination, and tube agglutination tests, sometimes accompanied with the use of 2-mercaptoethanol to distinguish IgG from IgM antibodies when determining the presence of active infection requiring antibiotic therapy; newer data obtained using genome-level screens suggest the potential utility of recombinant *B. melitensis* proteins for characterization of human infection [22]–[24]. Sometimes, when prozone or other interfering immune phenomena occur where clinical brucellosis may be associated with non-agglutinating antibodies, the Coomb's indirect antibody test or the BrucellaCapt assay can detect anti-*Brucella* antibodies [19], [25]–[32]. ELISA to detect IgM or IgG antibodies that react with *B. abortus* lysates are not recommended for diagnosis because of limited specificity, but a competitive ELISA to detect smooth *Brucella* LPS [33] and a rapid antibody-detecting test such as the lipopolysaccharide (LPS)-based lateral flow assay has favorable performance characteristics [19], [25]–[32]. Nonetheless, ELISA tests based on whole cell *B. abortus* lysates may suffer from false positive results. False positive serological results may be also found with other pathogenic bacteria because of cross-reaction with *E. coli* O157:H7, *Francisella tularensis*, *Yersinia enterocolica* and *Salmonella typhi* (at low dilutions) can confound serological diagnosis but diseases caused by these agents are rarely confused with brucellosis [34]–[39]. Nonetheless, serological diagnosis provides only an indirect measure of infection.

The present investigation aimed to develop new monoclonal antibodies against the immunodominant LPS of *B. melitensis,* towards the development of new tools for the direct detection of *Brucella* LPS antigen for diagnostic purposes. We adopted a new approach to enhance the affinity of IgG antibodies for the LPS antigen by coupling purified *B. melitensis* LPS to keyhole limpet hemocyanin (KLH) prior to immunization and boosting, which would be predicted to induce T cell-dependent affinity maturation of the anti-LPS antibody response by B cells. Supernatants from hybridomas which screened positive for anti-*B. melitensis* LPS by ELISA were further characterized by Western blot and indirect immunofluorescence microscopy. A capture ELISA using the purified monoclonal antibodies was tested for its ability to detect *B. melitensis* LPS antigen in sera from experimentally infected mice and Peruvian patients diagnosed with brucellosis by blood culture.

## Methods

### Purification of *Brucella melitensis* lipopolysaccharide

*Brucella melitensis* 16M was grown under aeration to stationary phase at 37°C in tryptic soy broth (TSB). Cells were recovered by centrifugation and approximately 10 g of pelleted cells were inactivated by autoclaving (121°C, 40 min). Cell pellets were used to isolate lipopolysaccharide (LPS) using the hot phenol-water method [40]. All manipulations of live *Brucella melitensis* were performed under Biosafety Level 3 conditions approved by the Select Agent program carried out at the University of California, Davis under conditions established and supervised by the United States Department of Agriculture and the United States Centers for Disease Control and Prevention. The purified LPSs were treated with RNase, DNase and proteinase-K (Sigma Chemicals, St. Louis, MO), and the hot phenol-water treatment was repeated. *B. melitensis* LPS fractions obtained from both upper phenol saturated aqueous layer (aqueous phase) and lower water saturated phenol layer (phenol phase) were pooled. Purified LPS was analyzed on a 4–12% gradient Tris-Glycine sodium dodecyl sulfate (SDS) polyacrylamide gel (Invitrogen Corp., Carlsbad, CA) under reducing conditions. The presence of LPS in the gels was detected with a periodic acid silver stain [41] and protein with Coomassie blue stain (BioRad, Hercules, CA). *B. melitensis* LPS was quantified using a colorimetric assay to measure 2-keto-3-deoxyoctonate (KDO) concentration [42]. *E. coli* 055: B5 LPS (Sigma Chemicals) was used as standard. We also extracted LPS from other Brucella species (*B. abortus*, *B. suis*, *B. canis*, *B. ovis*), *Yersinia enterocolitica 09* and *B. melitensis manB mutant* by following the earlier mentioned procedure.

### Keyhole limpet hemocyanin (KLH) conjugation to *B. melitensis* LPS and monoclonal antibody development

Three mg of purified *B. melitensis* LPS was used for conjugation with KLH (SoluLink, Inc., San Diego, CA). Briefly, periodate-treated *B. melitensis* 16M LPS was linked to succinimidyl 4-hydrazinonicotinate (SANH)-modified KLH. To estimate conjugation efficiency, KLH-conjugated *B. melitensis* LPS was analyzed on 10% Bis-Tris SDS PAGE gels (Invitrogen Corp., Carlsbad, U.S.A) under reducing conditions and stained with periodic acid silver that detects LPS.

Monoclonal antibodies were raised against KLH-conjugated *B. melitensis* LPS using standard methods [43], [44]. The primary screen for hybridoma supernatants was an ELISA using wells coated with *B. melitensis* LPS. Positive hybridoma supernatants were isotyped and IgG isotypes chosen for further study (Monoclonal Antibody Isotyping Kit, Pierce, Rockford, IL).

### LPS immunoblot analysis

To determine the reactivity of monoclonal antibodies to *B. melitensis* LPS, an immunoblotting analysis was performed as described previously [45]. *B. melitensis* LPS was electrophoresed on a 4–12% gradient Tris-Glycine SDS polyacrylamide gel and transferred to a nitrocellulose membrane using standard methods. The blotted membrane was blocked (Superblock, Thermo Fisher Scientific, Lake Barrington, IL) and strips were incubated individually in neat hybridoma supernatants for 2 h at room temperature. The strips were washed three times in TBS-Tween 20, 0.05% (TBST) and incubated with 1∶3000 dilution (1% Superblock in TBST) of goat anti-mouse IgG + IgM phosphatase labeled antibodies (KPL, Maryland, U.S.A) for 1 h and washed four times in TBST. The blots were incubated in substrate solution (BCIP/NBT-1 Phosphatase Substrate, KPL) for the color development.

To evaluate the cross reactivity of monoclonals, LPS of other Brucella species (B. abortus 2308 and B. suis 1330) and mutant (*B. melitensis* manB), E. coli 0157:H7 and *Y. enterocolitica* were electrophoresed, transferred and probed with hybridoma supernatants as described above. The dilution of the hybridoma supernatant varies between from 1∶50 to 1∶ 10,000) based on the signal intensity and it's mentioned in the blot for each monoclonal antibodies.

### Specificity of monoclonal antibodies against *B. melitensis* LPS by indirect immunofluorescence microscopy

ELISA-positive hybridoma supernatants were further screened by immunofluorescence against acetone-fixed *B. melitensis* 16 M, *E. coli* 0157:H7 strain EDL933 and *E. coli* strain DH10B expressing *Yersinia enterocolitica* 09 LPS [46]. Hybridoma supernatants were diluted 2-fold from 1∶50 to 1∶64,000; 50 µl of diluted supernatant was added to the acetone-fixed specimen and incubated for 2 h in a humidified chamber at 22°C. After three washes, FITC-labeled rabbit anti-mouse IgG and IgM (Jackson ImmunoResearch Laboratories, Inc.) was added at a 1∶100 dilution and incubated for 1 h at room temperature. After additional washes, the specimens were mounted with mounting medium (Invitrogen Corp., Carlsbad, CA), counterstained with DAPI (0.5 µg/ml; Molecular Probes, Inc.) and slides were examined using an Axiovert M200 microscope (Carl Zeiss, Germany).

### ELISA with whole cell antigen of *B. abortus* and *B. melitensis*

To determine the relative reactivity of mAbs for *B. abortus* and *B. melitensis*, a native whole cell antigen ELISA was used. Whole cell antigen was prepared by growth of *B. abortus* 2308 and *B. melitensis* 16M in tryptic soy broth with aeration at 37C for 16 h. Bacteria were diluted to an OD_600_ of 0.75, and 0.1 ml of antigen was incubated in a U-bottom microtiter plate with serial dilutions of mAbs for 1.5 h with rocking at 37°C. Three washes were performed by centrifugation (1000×*g*, 15 min) and resuspension in 0.1 ml PBS. After the last wash, bacterial pellets were suspended in a 1∶250 dilution of goat anti-mouse HRP conjugate and incubated 1.5 h with rocking at 37°C. After three washes, pellets were resuspended in HRP substrate and incubated at room temperature to allow for color development. After stopping the enzyme reaction, samples were filtered through 0.2 uM filters (MultiScreen HTS, Millipore) to eliminate bacteria and read at 450 nm. Data shown are from a single experiment that was replicated once with a similar result.

### Reactivity of *B. melitensis* LPS-reactive monoclonal antibodies by ELISA

An ELISA method was used to compare binding efficiency of mAbs according to a published method [47]. LPS from *E. coli* 055:B5 (Sigma) and *Leptospira licerasiae* [48] were used as specificity controls. *B. melitensis* LPS was dissolved (2 µg/ml) in 0.06 M sodium carbonate buffer (pH 9.6); 100 µl was used to coat wells by incubation overnight at 4°C. For the assay, Superblock blocking buffer (Thermo Fisher Scientific, IL, and USA) was used for blocking, TBST was used for washing the plate and 1% Superblock blocking buffer in TBST was used to dilute antibodies and conjugates. LPS-coated plates were blocked (for 1 hr at 22°C followed by three washes. mAbs were diluted (2 µg–0.02/ml) and added 200 µl per well in duplicate and incubated overnight at 4°C. The wells were washed three times with TBST and 200 µl of diluted (1∶5,000) goat anti-mouse immunoglobulin G (IgG) antibody conjugated to horseradish peroxidase was added to each well and incubated for 1 hr at room temperature. The plate was washed four times and 100 µl of chromogenic substrate (TMB Microwell Peroxidase Substrate system, KPL, Gaithersburg, MD) was added to each well and the reaction was stopped by the addition of 100 µl of 2N H_2_SO_4_. The plate was read using a microplate reader (Spectramax Plus, Molecular Devices, Sunnyvale, CA) at wavelength 450 nm.

### *B. melitensis* LPS antigen capture ELISA development in sera from experimentally infected mice

A total of 15 C57BL/6/c mice (Jackson Laboratories, Bar Harbor, Maine) were inoculated intraperitoneally with 2.8×10^5^ CFU of *B. melitensis* 16M. At weeks 1, 3 and 8 after infection groups of 5 mice were euthanized and blood collected at necropsy. Colonization of liver and spleen was determined by serial dilution of tissue homogenates and plating on tryptic soy agar (TSA). These experiments were carried out under ABSL-3 containment at UC Davis and approved by the UC Davis Institutional Animal Care and Use Committee. As negative control, sera from uninfected mice (N = 3) were obtained. Serum samples were diluted 1∶5 in phosphate-buffered saline and were passed twice through 0.22 µM filters for biological safety reasons. All samples were streaked on TSA + blood plates to confirm a lack of viable bacteria before testing for antigen by ELISA.

### *B. melitensis* LPS antigen capture ELISA testing on human sera

Two *B. melitensis* LPS immuno-reactive mAbs were used for assay development: 2D1 mAb as capture antibody (200 ng/well) and biotinylated 2E8 (0.5 mg/ml) for detection. Biotinylation of 2E8 mAb was carried using standard methods according to manufacturer's instructions (EZ-Link Biotinylation, Thermo Scientific, Ill, U.S.A). *B. melitensis* LPS antigen detection was carried out using: a) *B. melitensis* LPS spiked into the human brucellosis case and control sera, b) *B. melitensis* infected and control mice sera and c) Sera from patients with brucellosis confirmed by blood culture to have *B. melitensis* infection and sera from control patients from Peru who were referred to the study for suspected brucellosis but were found to be blood culture and serologically negative for brucellosis [23], [24]. The available serological standard tests (Rose Bengal test, lateral flow assay [29] were used to screen patients for brucellosis. Routine diagnostic tests for brucellosis confirmation included the standard tube agglutination assay and 2-mercaptoethanol test (to distinguish IgM and IgG agglutinating antibodies) and were also carried out on this set of patient samples, as previously described [23], [24], [32]. A commercially available ELISA test kit, based on a *B. abortus* whole cell lysate putatively used for brucellosis, was also used (Genway IgG Brucellosis ELISA, Catalog GWB-3C0D26, San Diego, CA).

Normal human sera (N = 10) purchased from a commercial blood bank in USA (Interstate Blood Bank, Memphis, TN) were spiked with measured quantities of *B. melitensis* LPS for testing in the capture assay; unspiked sera served as negative controls. Sera from human brucellosis cases were obtained at the time of blood culture positivity; 10 negative control sera from Peru (culture negative and Rose Bengal Test negative) were used.

The antigen-detection ELISA was developed by coating plates with capture mAb 2D1 (200 ng/well) in 0.06 M sodium bicarbonate buffer (pH 9.6) incubated overnight at 4°C in a humid chamber. Blocking and washing steps, antibody dilution buffer and color development were done as described above. Test serum samples were diluted (1∶25 dilution) in 1% Superblock TBST and 200 µl of the diluted sample was added per well in duplicates and incubated over night at 4°C. For human serum samples from Peru, the dilution was done in TBST and heat inactivated (56°C for 15 minutes) before adding to the wells. The plates were washed after incubation in test serum samples. For detection, 200 µl of biotinylated mAb 2E8 (1∶5,000 dilution, 0.5 mg/ml) was added per well and incubated for 2 hr. Plates were washed 4 times and 200 µl of 1∶8,000 dilution of peroxidase-conjugated streptavidin (Thermo Scientific, Ill, USA) was added and incubated for one hr. The plates were washed and colorimetric assay developed as described above.

### Ethics statement

Human subjects participating in this study and providing clinical samples (blood only) for routine and new experimental approaches to brucellosis diagnosis were part of a prospective study to develop brucellosis diagnostics based in Lima, Peru from 2005–2010. The enrollment of human subjects in this study was carried out under a protocol approved by the Human Subjects Protections Program, University of California San Diego and the Ethical Committee of Universidad Peruana Cayetano Heredia, Lima, Peru. The use of the samples for the purposes described in the present experiments was approved by both Institutional Review Boards. All samples were anonymized before sending for analysis. All adults provided written informed consent to participate in this study; all minors involved in the study provided verbal assent and written informed consent was provided by each minor's parent/guardian.

### Data analysis

Data analysis and graphs were done with Prism 5.0 software (GraphPad Prism, San Diego, CA). For statistical significance between positive and control groups, Student's t test was performed with *p*<0.05 considered statistically significant. ELISA results were considered positive when the O.D. was ≥3.0 standard deviations above the mean of the negative control (in triplicate).

## Results

### Production and selection of hybridomas producing anti-*B. melitensis* monoclonal antibodies

**Figure 1:**
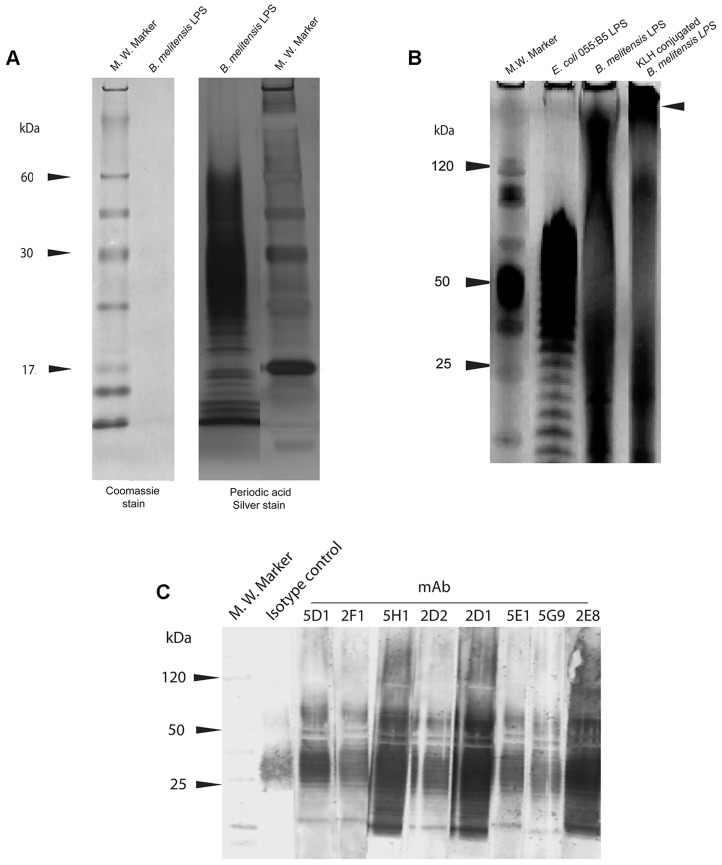
Sodium dodecyl sulfate polyacrylamide gel electrophoresis and western immunoblot analysis of purified and conjugated *Brucella melitensis* lipopolysaccharide. A. *B. melitensis* LPS was separated on a 4–12% Tris Glycine SDS-polyacrylamide gradient gel. The gel was stained for protein contamination of the lipopolysaccharide preparation with Coomassie Blue and periodic acid silver stain for LPS detection. Molecular mass (kDa) indicated at right of the panel. B. Keyhole Limpet hemocyanin (KLH) conjugated to *B. melitensis* LPS. *B. melitenis* LPS was oxidized with sodium periodate and linked to succinimidyl 4-hydrazinonicotinate (SANH)-modified KLH. *Escherichia coli* O55:B5 LPS (control), *B. melitensis* LPS alone and KLH-conjugated- KLH-conjugated *B. melitensis* LPS. The samples were electrophoresed on a 10% Bis-Tris polyacrylamide gel and stained with silver stain for LPS detection. Molecular mass (kDa) indicated at left of panel. C. Western immunoblot of anti-LPS monoclonal antibodies. Purified *B. melitensis* LPS was subjected to SDS-PAGE, transferred to nitrocellulose, and strips were probed individually with hybridoma supernatants (mAbs: 5D1, 2F1, 5H1, 2D2, 2D1 and 5E1).

Mice vaccinated with purified, protein-free *B. melitensis* LPS (Fig. 1A) conjugated to keyhole limpet hemocyanin (KLH) (Fig. 1B) were sacrificed, splenocytes fused to non-secreting myeloma cells and hybridomas screened by ELISA for reactivity against *B. melitensis* LPS. Eight hybridomas screening positive by ELISA were confirmed to recognized *B. melitensis* LPS by Western immunoblot (Fig. 1C).

### Specificity of anti-*B. melitensis* 16M LPS monoclonal antibodies using western immunoblot, ELISA and indirect immunofluorescence assay

**Figure 2:**
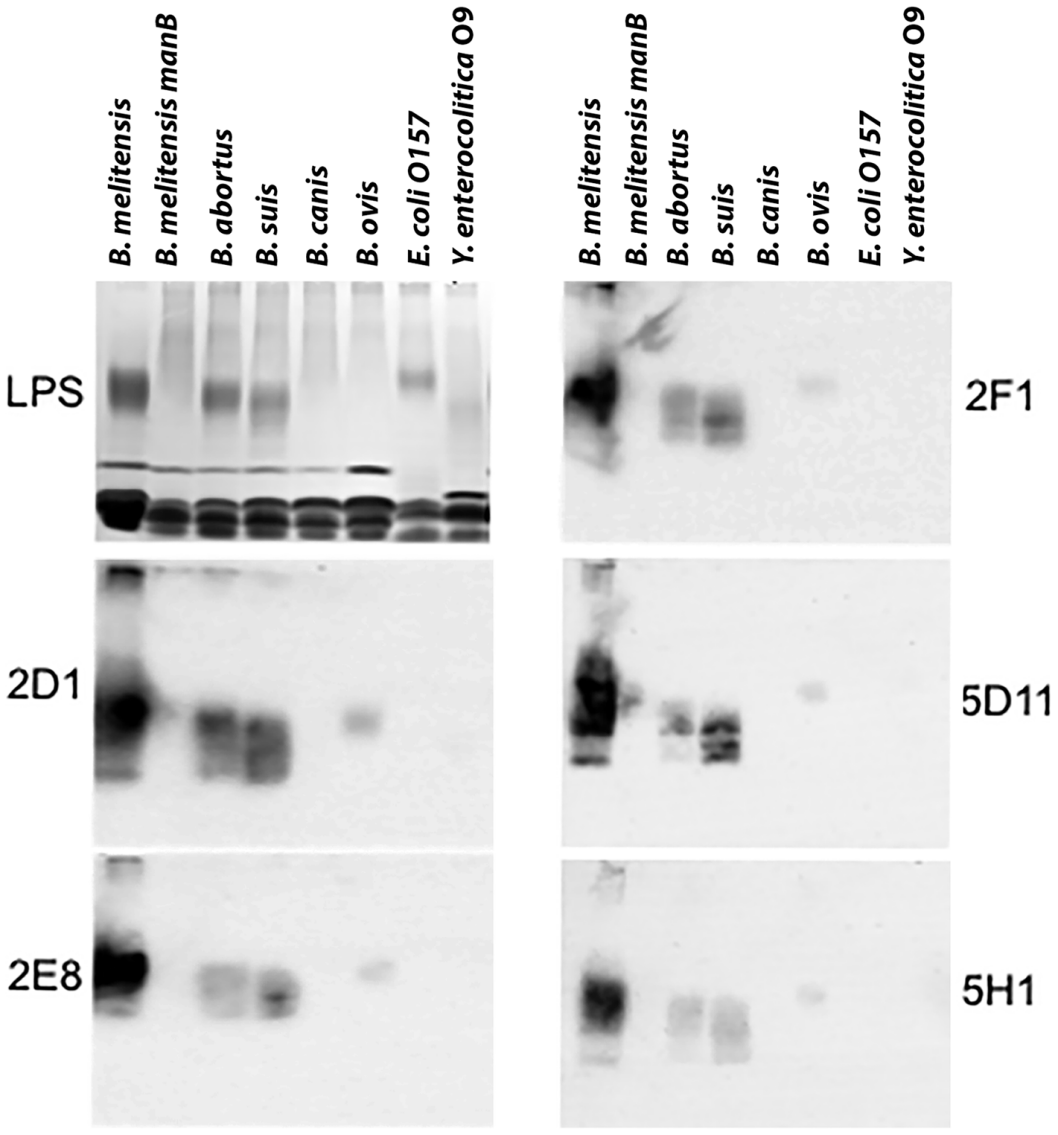
LPS immunoblot probed with monoclonal antibody-containing supernatants to determine cross reactivity to the main pathogenic species of *Brucella* and potentially cross-reactive organisms. Upper left, silver stain for LPS. Remaining panels reacted with mAbs as indicated.

The reactivity of six monoclonal antibodies was determined against LPS antigens from *B. melitensis* strain 16M (genome strain), a mutant LPS-deficient *B. melitensis* 16M *manB* mutant [49], *B. abortus, B. suis, B. canis, B. ovis, E. coli* O157:H7, and *Yersinia enterocolitica.* LPS extracts were electrophoresed on a 10% Bis-Tris polyacrylamide gel and stained with silver stain for LPS detection (LPS)(Fig. 2, upper left panel). Other gels were transferred to nitrocellulose, and strips were probed individually with dilutions of hybridoma supernatants as indicated: 2D1 (1∶1,000), 2E8 (1∶10,000), 2F1 (1∶100), 5D1 (1∶500) and 5H1 (1∶5,000). The mAbs reacted strongly with *B. melitensis* 16M (but not the LPS-deficient *manB* mutant), and with *B. abortus, B. suis,* and, faintly, with *B. ovis.* No cross reactivity was observed by Western immunoblot with *E. coli* O157:H7 or *Y. enterocolitica.*

**Figure 3:**
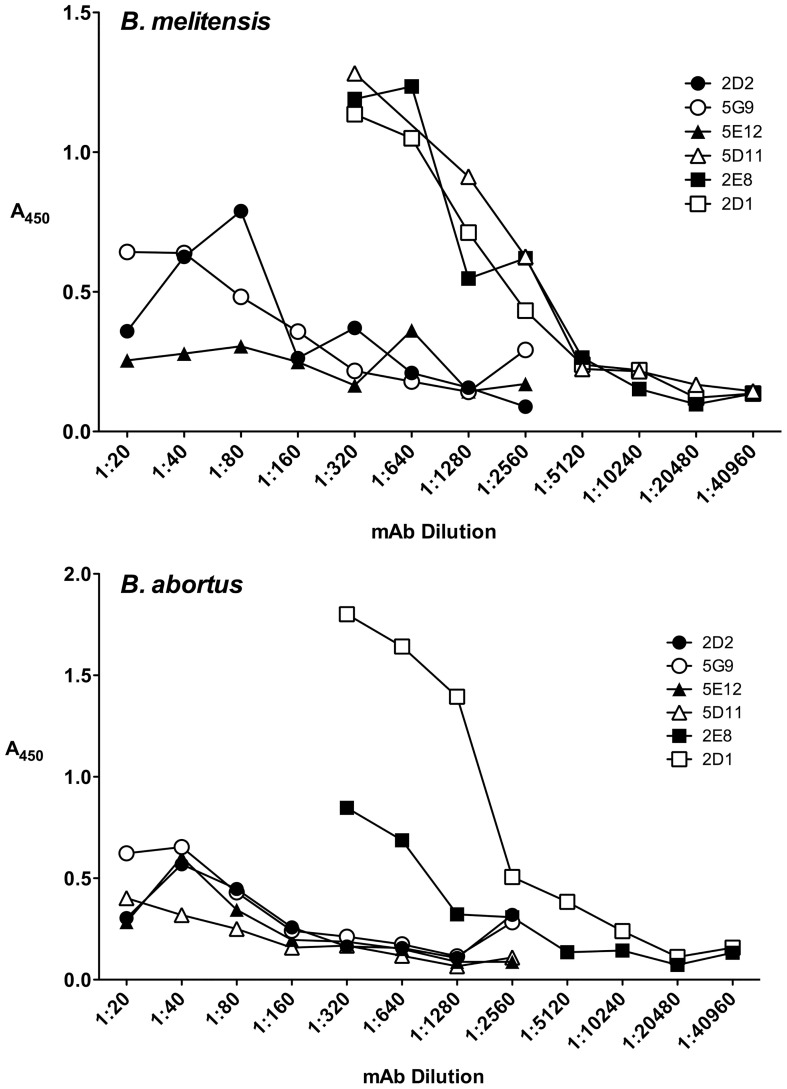
ELISA-determined reactivity of a panel of monoclonal antibodies raised against KLH-conjugated *B. melitensis* 16M lipopolysaccharide against *B. melitensis* 16M and *B. abortus* 2308 native whole cell antigen.

To further investigate potential cross-reactivity using a different immunochemical technique, soluble antigens from *B. melitensis* 16M and *B. abortus* 2308 using an ELISA (Fig. 3A and B). Five of the 6 demonstrated reactivity against both *Brucella* spp.; one, 5D11 produced a detectable reaction with *B. melitensis* 16M but not with *B. abortus* 2308 (bottom right panel, Fig. 3). To characterize further the binding specificity of these mAbs using native (not heated, not chemically extracted) LPS, indirect immunofluorescence microscopy (IFA) was carried out on *B. melitensis* and *B. abortus* fixed directly onto slides (Table 1); in addition, *E. coli* O157:H7 strain EDL933 and *E. coli* strain DH108 expressing the *Yersinia enterocolitica* O9 antigen previously reported to cross-react with *Brucella* LPS [37], [38] were used as antigen for IFA (Table 1). Based on shared patterns of reactivity for both *B. melitensis* 16M and *B. abortus* 2308, mAbs 2D1 and 2E8 were chosen for further development of a *Brucella* LPS antigen capture ELISA.

**Table 1:** Specificity of anti-*Brucella melitensis* 16M lipopolysaccharide monoclonal antibodies by indirect immunofluorescence assay.

Species/Strain	mAb Dilution*	Monoclonal Antibody							
		2D1	2E8	5H1	2D2	5G9	5D1	2F1	5E1
*B. melitensis* 16M	1:50	+	+	+	Weak +	+	+	+	+
	1:100	-	+	-	Weak +	+	+	-	+
	1:200	-	-	-	Weak +	+	+	-	-
	1:400	-	-	-	-	-	+	-	-
	1:800	-	-	-	-	-	+	-	-
	1:1600	-	-	-	-	-	+	-	-
	1:3200	-	-	-	-	-	+	-	-
	1:6400	-	-	-	-	-	+	-	-
*E. coli* 0157:H7 strain EDL933	1:50	-	+	+	-	-	-	-	+
*E. coli* expressing *Yersinia enterocolitica* O9 LPS (*E. coli* strain DH10B)	1:50	+	-	+	-	+	+	-	+

*Hybridoma supernatants were used at the dilutions indicated. mAb, monoclonal antibody.

### Development of an antigen capture ELISA using anti-*Brucella melitensis* 16M LPS monoclonal antibodies

**Figure 4:**
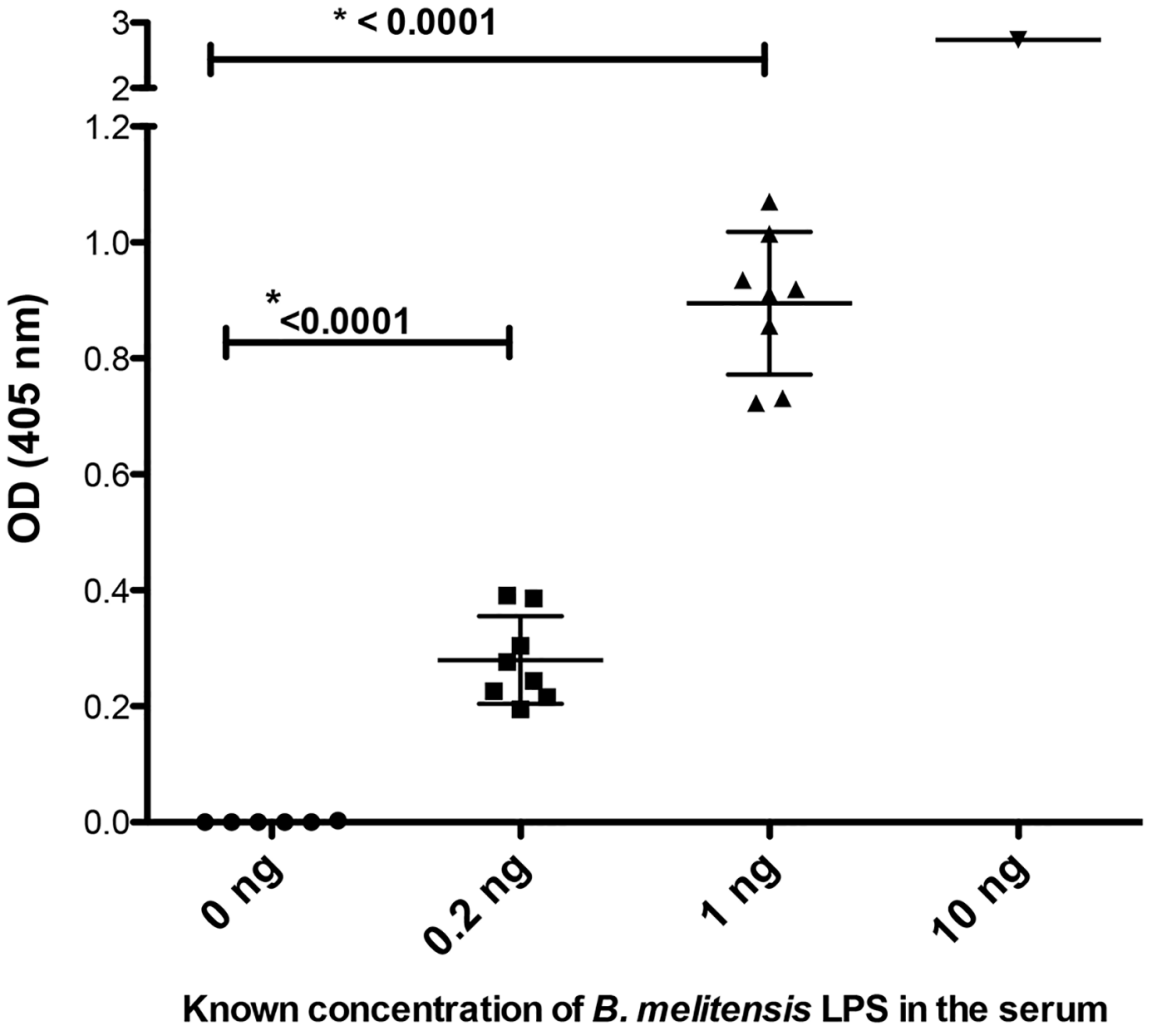
Sensitivity of *B. melitensis* LPS antigen detection by capture ELISA. Monoclonal antibody 2D1 to capture antigen and biotinylated mAb 2E8 used to detect LPS in the human serum samples that were spiked with known amount of *B. melitensis* LPS. The average absorption value obtained from the duplicate wells were plotted in the Y-axis, and the mean value along with standard deviation in for each groups were represented as bar. Positive result defined as OD_450_ ≥3.0 standard deviations from mean value of negative control group (0 ng LPS). Capture ELISA using *Escherichia coli* 055:B5 LPS (10 ng) and *Leptospira licerasiae* LPS (10 ng) did not yield a signal above background and not shown in the figure.

MAbs 2D1 (capture antibody) and 2E8 (biotinylated for detection) were tested in a checkerboard assay for optimum concentrations for the development of a capture ELISA to detect *B. melitensis* 16M LPS (data not shown). A capture ELISA using these mAbs specifically detected *B. melitensis* 16M LPS spiked into normal human serum (Fig. 4), not producing a signal with *E. coli* or *Leptospira licerasiae* LPS.

### Determination of *B. melitensis* LPS antigenemia in experimentally infected mice

**Figure 5:**
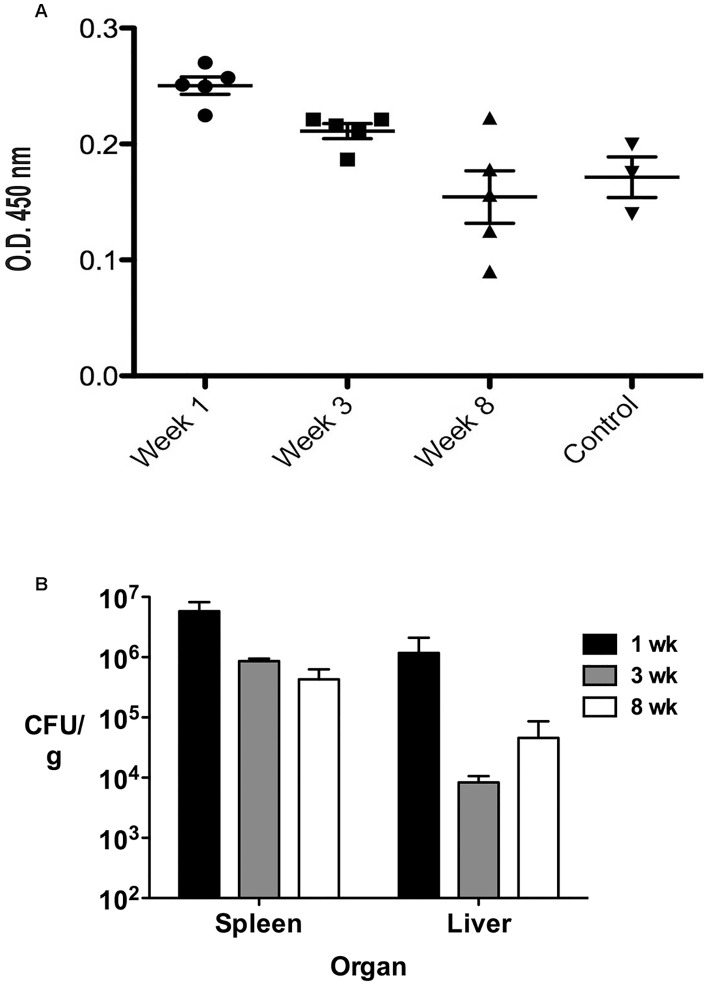
Detection of *B. melitensis* LPS in serum of experimentally infected mice. A. BALB/c mice were infected with *B. melitensis* 16M. Sera were tested for the presence of LPS at weeks 1, 3 and 8 using the 2D1-2E8 capture ELISA. *B. melitensis* LPS was detected above background at weeks 1 (*p*≤0.003) and 3 (*p≤*0.004) but not at week 8. B. Colony forming units (CFU) in liver and spleen at each time point are indicated.

To determine whether the capture assay would be able to detect *B. melitensis* LPS in serum of experimentally infected mice, C57BL/6 mice were injected with 10^6^ CFU of a low passage strain of *B. melitensis* 16M, and serum, liver and spleens obtained at 1,3 and 8 weeks after inoculation. *B. melitensis* LPS was detected at a significantly higher level in serum at 1 and 3 weeks post-infection but was not detectable at 8 weeks, despite persisting viable *B. melitensis* observable in liver and spleen as determined by CFU counts (Fig.5, bottom).

### Determination of *B. melitensis* LPS antigenemia in Peruvian brucellosis patients

**Figure 6:**
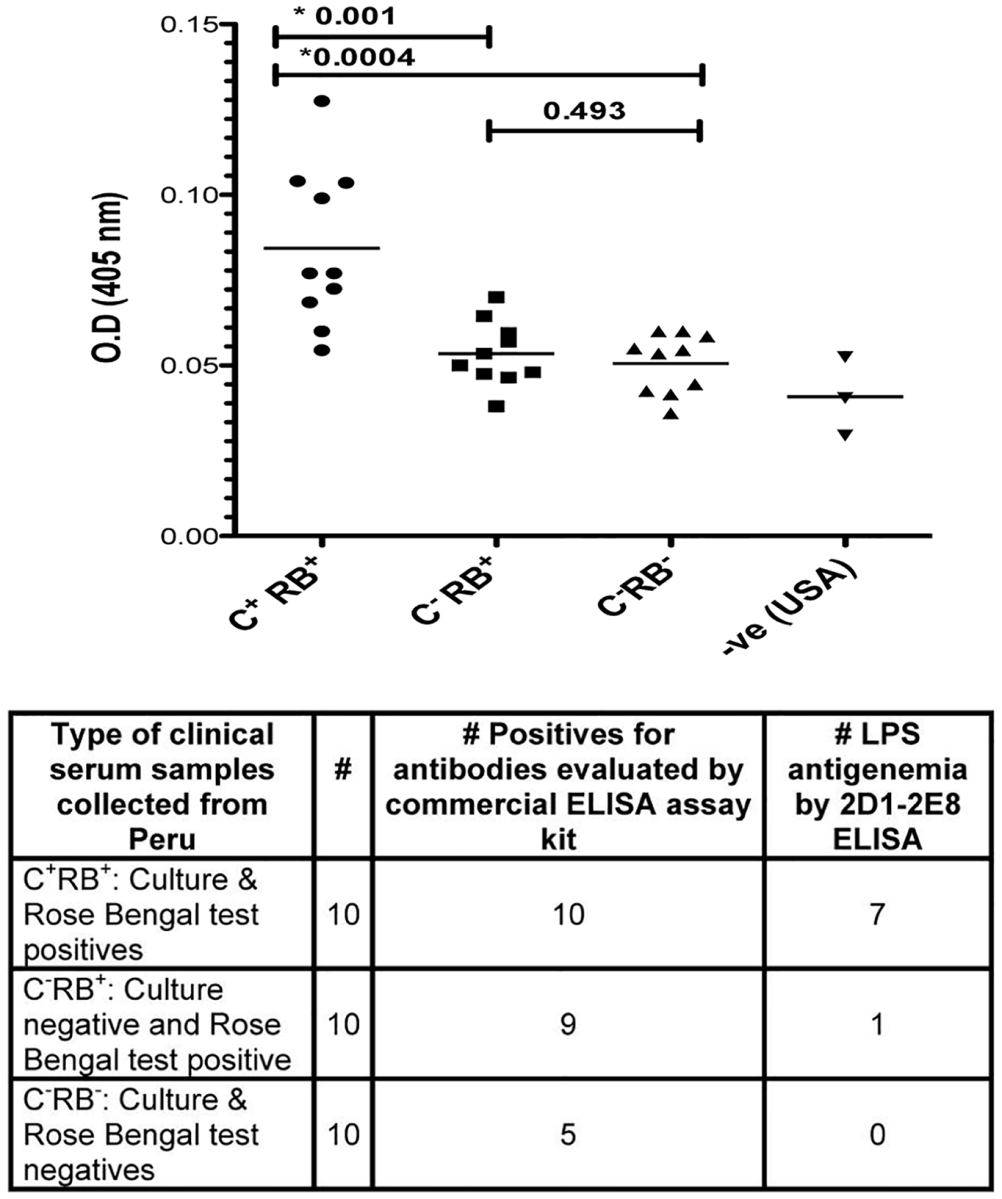
Detection of *B. melitensis* LPS in human brucellosis patients from Peru. Sera from patients presenting with a suspicion of brucellosis were either confirmed to have *B. melitensis* brucellosis by blood culture and serological positive (C^+^RB^+^); serologically positive but blood culture negative (C^-^ RB^+^) and both serologically and blood culture negative samples (C^-^ and RB^-^). Three negative control serum obtained from US blood bank (-ve USA) were also included in the assay. The average absorption value of the samples obtained by capture ELISA were plotted, and the mean value in each group are shown as bar. The signal was significantly higher in the blood culture positive than in the blood culture negative/serologically negative group (*p*≤0.0004) and blood culture negative/seropositive group (*p*≤0.001). The lower table shows the comparative result by serological tests (Rose Bengal test and Brucella IgG ELISA Kit, GenWay Biotech Inc, San Diego, CA) and the capture ELISA, which shows 70% (7/10) of the culture positive samples were detected with higher LPS antigenemia (OD_450_≥3.0 standard deviations from the mean value of six Peruvian C^−^S^−^ samples).

Ultimately, the capture ELISA to detect *B. melitensis* LPS was developed with the goal of having a culture-independent method of rapidly diagnosing brucellosis. In a pilot study of 30 patients referred for suspected brucellosis, we studied three groups of 10 subjects each (Table 2): 1) with definitive brucellosis (blood culture positive); 2) Rose Bengal screen positive, and various titers of standard tube agglutination positive patients but negative blood cultures; and 3) serologically and blood culture negative. A majority (7/10) of the patients with positive blood cultures had *B. melitensis* LPS detected in the initial blood specimen obtained (Table 2, Figure 6). One of ten patients (with relapsed brucellosis) with a negative blood culture had a positive serum antigen test. No seronegative and blood culture negative patient had a positive serum antigen test.

**Table 2:** Clinical features and diagnostic results of Peruvian patient groups.

Sample Number	Clinical Syndrome	Blood Culture (Days to +)	Rose Bengal	IgM LFA	IgG LFA	sTAT Titer	2-ME Titer	GenWay IgG test (antibody index#)
1	Acute	+ (d 10)	+	2	2	160	0	+ (3.312)
2	Acute	+ (d 12)	+	4	0	320	0	+ (3.328)
3	Acute	+ (d 4)	+	4	0	320	0	+ (1.902)
4	Acute	+ (d 7)	+	4	0	320	0	+ (4.106)
5	Acute	+ (d 6)	+	4	2	320	20	+ (3.672)
6	Neuro-brucellosis Paraparesis, encephalitis	+ (d 6)	+	0	2	0	0	+ (5.888)
7	Acute	+ (d 6)	+	2	0	640	160	+ (2.964)
8	Acute	+ (d 9)	+	3	2	320	40	+ (3.598)
9	Acute	+ (d 6)	+	2	1	160	80	+ (5.996)
10	Acute	+ (d 7)	+	4	0	160	0	+ (2.618)
11	Acute, incomplete treatment	- ve	+	3	0	320	0	+ (3.476)
12	Acute	- ve	+	1	1	640	320	+ (3.928)
13	Acute	- ve	+	4	0	320	0	+ (6.304)
14	Family contact of acute brucellosis case, asymptomatic	- ve	+	4	0	320	0	+ (5.008)
15	Suspected brucellosis relapse	- ve	+	4	2	320	20	+ (1.212)
16	Four time brucellosis relapse diagnosed serologically	- ve	+	0	2	0	0	+ (6.276)
17	Acute	- ve	+	2	0	640	160	-ve (0.270)
18	Relapse, symptoms of arthralgia, general fatigue, depression, insomnia	- ve	+	2	1	320	20	+ (4.884)
19	Subacute onset of headache, sweating, prostartion, diarrhea	- ve	+	2	0	40	20	+ (3.3)
20	Evaluated 42 days after completing treatment, relapsed clinically 2 months later	- ve	+	3	2	320	80	+ (3.102)
21	Unconfirmed Brucellosis*	- ve	-ve	0	0	0	0	-ve (0.38)
22	Unconfirmed Brucellosis*	- ve	- ve	0	0	0	0	+ BL (0.962)
23	Unconfirmed Brucellosis*	- ve	-ve	0	0	0	0	+ (2.76)
24	Unconfirmed Brucellosis*	- ve	-ve	0	0	0	0	-ve (0.614)
25	Unconfirmed Brucellosis*	- ve	-ve	0	0	0	0	-ve (0.424)
26	Unconfirmed Brucellosis*	- ve	-ve	0	0	0	0	-ve (0.584)
27	Unconfirmed Brucellosis*	- ve	-ve	0	0	0	0	+ (1.64)
28	Unconfirmed Brucellosis*	- ve	-ve	0	0	25	0	-ve (0.586)
29	Unconfirmed Brucellosis*	- ve	-ve	0	0	0	0	+ (3.086)
30	Unconfirmed Brucellosis*	- ve	-ve	0	0	25	0	+ (1.276)

**LFA** (Lateral flow assay)**; sTAT** (Standard tube agglutination test); * Patient referred for suspicion of brucellosis by clinician but diagnosis not confirmed)**; 2-ME** (2-mercaptoethonal brucella agglutination test).

# Antibody index (AI) was calculated as per protocol (Brucella IgG ELISA, GenWay Inc, USA). The classification of AI are: <0.9 (No detectable Antibody), 0.9-1.1 (Border line positive or BL, follow up test recommended) and >1.1 detectable antibody.

## Discussion

Here we report the development of a monoclonal antibody-based *Brucella melitensis* antigen detection assay based on new monoclonal antibodies developed using a novel LPS-protein conjugation approach. Here we made *B. melitensis* LPS into a T-dependent antigen by chemical conjugation to a carrier protein, keyhole limpet hemocyanin (KLH) to enhance immunogenicity by inducing helper T cell function. Such an approach has been demonstrated to increase titers and affinities of antibody responses against T cell-independent antigens such as bacterial capsular polysaccharides [50]–[55] and the potential for enhancing affinity maturation of poorly antigenic proteins [56], [57] as well as T-independent antigens [58] such as LPS. The immunization strategy used here yielded a panel of monoclonal antibodies with varying properties. Hybridomas producing mAbs that recognized both *B. melitensis and B. abortus* LPS and one that produced a mAb that selectively recognized *B. melitensis* were obtained. An antigen capture assay was developed that was able to detect *B. melitensis* LPS in the serum of experimentally-infected mice and blood culture-positive brucellosis cases in Peru.

The present work is consistent with previous reports of mAb development against *Brucella* LPS in detecting both shared and unique epitopes (O-antigen, A- and M- epitopes) within *Brucella* spp. LPS [14], [59]–[63]. Using the new panel of mAbs, we found that in the mouse model of brucellosis that LPS antigen was detectable at 1 and 3 weeks after infection. However, while colony counts of *B. melitensis* remained robust in liver and spleen eight weeks after infection, LPS antigen was no longer detectable. This discrepancy may relate to the human data reported here in which we found only a moderate sensitivity (70%) of LPS antigen detection at clinical presentation and poor sensitivity (10%) when in seropositive, blood culture negative patients. This observation is consistent with the known imperfect sensitivity of blood culture for the diagnosis of brucellosis, and suggests the compartmentalization of *B. melitensis* during human infection into sites where neither bacteria nor LPS are released into blood after acute infection. We do not believe that the performance of the capture ELISA reported here is due to lack of performance of the mAbs for two reasons: 1) the mAbs used were determined to detect both *B. melitensis* and *B. abortus* LPS; and 2) human brucellosis in Peru is caused exclusively by *B. melitensis*, so that non-*B. melitensis* brucellosis cases are unlikely to have occurred in our patient population. It is possible that the intracellular location of *Brucella* may reduce the sensitivity of LPS antigen detection. Regarding the specificity of the mAb detection ELISA described here, the pair of mAbs—2D1 and 2E8—were chosen specifically not to detect either *E. coli* O157:H7 or *Yersinia enterocolitica*, which was confirmed by Western immunoblot. Regardless, these infections present quite differently than brucellosis and would not be confused by an experienced clinician; hence antigen detection assays for brucellosis would not likely yield false positive results in these disease. Indeed, it may be possible to use the relevant mAbs (Table 1) to detect infections by either of these organisms in the appropriate clinical context.

The antigen-detection mAbs described here also have a One Health application, and could readily be adapted for screening herds of livestock for active infection that present either animal or human health risk. The ability of this panel of mAbs to identify *B. suis* antigen was not assessed here; it would be unlikely that *B. canis* infections would be identified given the lack of LPS in this species. The assay here only was tested with *B. melitensis* infection, and further work needs to be done to assess which of the mAbs would be most effective to identify *B. abortus* infection.

Here we did not assess the ability of the mAb capture assay to detect persistent antigenemia to suggest persistent infection, which has been suggested by persistent DNAemia after *B. melitensis* infection [15]. We believe, however, that our data are consistent with the detection of active bacteremia and disease, which persistent DNAemia does not necessarily indicate. Chronic and occult brucellosis is difficult to diagnosis, likely due to low organism burden, hence we would not expect the mAb capture ELISA described here to be sensitive in diagnosing this manifestation of brucellosis. Nonetheless, the utility of antigen detection to diagnosis manifestations of brucellosis such as focal or chronic forms remains to be determined. Finally, antigen detection in specimens other than serum needs to be further assessed in other endemic settings and other clinical specimens such whole blot, blood clot, bone marrow, urine, abscess aspirates and cerebrospinal fluid.
